# Interference between Space and Time Estimations: From Behavior to Neurons

**DOI:** 10.3389/fnins.2017.00631

**Published:** 2017-11-21

**Authors:** Encarni Marcos, Aldo Genovesio

**Affiliations:** Department of Physiology and Pharmacology, Sapienza University of Rome, Rome, Italy

**Keywords:** monkeys, prefrontal cortex, time perception, working memory, magnitude processing

## Abstract

Influences between time and space can be found in our daily life in which we are surrounded by numerous spatial metaphors to refer to time. For instance, when we move files from one folder to another in our computer a horizontal line that grows from left to right informs us about the elapsed and remaining time to finish the procedure and, similarly, in our communication we use several spatial terms to refer to time. Although with some differences in the degree of interference, not only space has an influence on time but both magnitudes influence each other. Indeed, since our childhood our estimations of time are influenced by space even when space should be irrelevant and the same occurs when estimating space with time as distractor. Such interference between magnitudes has also been observed in monkeys even if they do not use language or computers, suggesting that the two magnitudes are tightly coupled beyond communication and technology. Imaging and lesion studies have indicated that same brain areas are involved during the processing of both magnitudes and have suggested that rather than coding the specific magnitude itself the brain represents them as abstract concepts. Recent neurophysiological studies in prefrontal cortex, however, have shown that the coding of absolute and relative space and time in this area is realized by independent groups of neurons. Interestingly, instead, a high overlap was observed in this same area in the coding of goal choices across tasks. These results suggest that rather than during perception or estimation of space and time the interference between the two magnitudes might occur, at least in the prefrontal cortex, in a subsequent phase in which the goal has to be chosen or the response provided.

Einstein's Theory of Relativity postulates that time and space are tightly coupled. This is, time passes in the same way for all non-accelerating observers, but differently for observers traveling at different speeds. Inspired by further questions formulated by Einstein, Piaget conducted a series of experiments about time and space with children. These experiments led him to claim that “time and space form an inseparable whole” (Piaget, 1927), although with some asymmetries in the way that one magnitude influences the other. Such asymmetries can be found in language (Clark, 1973; Traugott, 1978; Alverson, 1994) and co-speech gestures (Núñez et al., 2012). For instance, when using language, it is common to use words or concepts borrowed from the spatial domain. In English, expressions such as “long”, “short”, “move forward,” or “ahead of” are often used to refer to time. Moreover, children learn first spatial than temporal terms and indeed metaphorical expressions of spatial terms are used as temporal terms (Clark, 1973). Such metaphoric manner in which spatial terms are used to refer to time is not only found in linguistics but also in the graphical representation of time. In other words, specific spatial locations relative to a reference point are generally used to indicate either “past” or “future” events (Boroditsky, 2000; Torralbo et al., 2006). These findings have led to metaphorical theories of mental representation that propose that time and space influence each other in an asymmetrical way (Lakoff and Johnson, 1999).

Recent studies have investigated whether the dependency between the two magnitudes also exists when we merely think about them. Indeed, it has been observed that the link between time and space is not uniquely constraint to communication but it is also present when estimating duration and dimension of different stimuli (Xuan et al., 2007; Casasanto and Boroditsky, 2008) or when estimating the passage of time in spatial environments of different sizes (De Long, 1981; Mitchell and Davis, 1987). Moreover, brain lesions affecting spatial processing also influence time estimation (Basso et al., 1996) whereas, on the contrary, people with synesthesia exhibit enhanced magnitude processing (Teuscher et al., 2010; Cohen Kadosh et al., 2011). In addition, the discrimination of time, space, and other magnitudes, obeys Weber-Fechner's law (Stevens and Greenbaum, 1966; Teghtsoonian and Teghtsoonian, 1978) according to which the perceived change in the magnitude of a stimulus is proportional to its initial value of magnitude. Based on this experimental evidence, a Theory of Magnitude (ATOM) proposes that a general neural mechanism is responsible for the computation of magnitudes (Walsh, 2003). According to ATOM, neurons encode, for instance, time and space in an abstract manner independently of the specific magnitude. Besides behavioral evidence, this theory has been also supported by lesion studies showing time and space processing impairments (Critchley, 1953; Basso et al., 1996). However, more recently, independent groups of neurons with a domain-specific coding of magnitude have been found in the prefrontal cortex (Genovesio et al., 2012; Marcos et al., 2017).

## Interference between space and time in behavior

We use words from concrete domains to refer and to think about abstract domains because concrete domains are the ones that we can really experience. Such cross-domain use of semantic terms can be found in our language and gestures when we refer to love or time among other abstract concepts (Lakoff and Johnson, 1980; Lakoff and Kovecses, 1987; McNeill, 1992). For instance, metaphoric expressions such as, “Winter is coming” (see *Games of Thrones*) or “The trip was short” are often used to express temporal concepts and they shape our idea of time through spatial metaphors. This occurs because we can perceive the concrete but only imagine the abstract (Evans, 2004). For instance, through experience, we learn about the spatial domain because we can perceive the distance between two points or the relative position of one object respect to another. Then, we import terms and schemas from this domain to structure our idea of time and to materialize what we have learned, also through experience, about time, such as, that time always moves forward and that all days have a beginning and an end.

Using non-linguistic tasks, psychophysics experiments have examined the influence that time and space have on each other (Casasanto and Boroditsky, 2008; Casasanto et al., 2010; Merritt et al., 2010; Mendez et al., 2011). Casasanto and Boroditsky (2008) tested the ability of humans to reproduce either the duration or the spatial displacement of lines or dots displayed on a screen. Both magnitudes were always presented and one of them served as the testing variable while the other was simply a distractor. Participants' estimations of duration were influenced by the spatial displacement but not vice versa. Moreover, the effect was not related to a difference in accuracy between duration or displacement estimates and was also observed when participants were given prior knowledge about the magnitude to attend to. Interestingly, spatial influence on duration estimates was sensory independent and also present when the duration of the stimuli was provided through visual and auditory modalities. Thus, the effect of space on time was robust across the different experimental manipulations. Moreover, similar to linguistic metaphors, the results suggest that mental representations of time rely on mental representations of space.

A subsequent study examined whether the mental representation of time in adults emerges after cognitive development or whether it already exists in young children (Casasanto et al., 2010). To do that, the authors ran a series of experiments in children aged from 4 to 6 and from 9 to 10 years old. The experiments were analogous to those in adults (Casasanto and Boroditsky, 2008). Although children's ability to judge temporal and space magnitudes was equivalent they were more influenced by the spatial components during temporal estimations than vice versa. This result confirms that children exhibit the same asymmetry found in adults for estimations of time and space. Therefore, together with previous studies, the dependency between time and space in non-linguistic tasks in humans seems to be asymmetrical, similar to the influence observed during communication.

## Shared mechanism for magnitude estimations

Some behavioral mechanisms are common to several magnitude estimations. For instance, independently of whether subjects are estimating time, distance, or length, there is a tendency, that increases for large sample ranges, to estimate values which are biased toward the mean of the distribution (Hollingworth, 1910; Stevens and Greenbaum, 1966; Teghtsoonian and Teghtsoonian, 1978; Jazayeri and Shadlen, 2010; Petzschner and Glasauer, 2011). Bayesian models have explained such general behavioral phenomena as the result of integrating prior experience and noisy sensory information (Jazayeri and Shadlen, 2010; Petzschner and Glasauer, 2011). Likewise, magnitude sensitivity is similar in different domains using time, space, and number tasks (Hauser et al., 2000; Brannon et al., 2006; Halberda et al., 2006; Beran, 2007). Additionally, a close connection between numbers and space has been observed, in which numbers can have a continuous spatial representation organized along a “mental number line” with, at least in Western culture, small numbers on the left side of space and large numbers on the right (Restle, 1970; Dehaene et al., 1990, 2003a; Hubbard et al., 2005). Numerical processing can cause shifts in covert attention as a function of their magnitude (Casarotti et al., 2007; Dodd et al., 2008; Nicholls et al., 2008), influence motor planning leading to faster responses toward left for small numbers and toward right for big numbers (Fischer, 2003; Fischer et al., 2004) and impact high-level cognitive processes such as, non-propositional reasoning (Brunamonti et al., 2011). On the other hand, visuospatial variables, such as, spatial cueing or visual hemifield presentations, can also influence numerical comparisons (Lavidor et al., 2004; Nicholls and McIlroy, 2010). All these experimental evidence have led to the idea of ATOM that proposes that there is a common processing mechanism for magnitudes.

Functional neuroimaging, neuropsychological, and neurophysiological studies (Critchley, 1953; Basso et al., 1996; Dehaene et al., 2003a; Fias et al., 2003) have also supported the theory of a general mechanism for magnitude processing. For instance, a Positron Emission Topography (PET) study in which monkeys were trained to perform a duration discrimination task (Onoe et al., 2001) showed that the dorsolateral prefrontal cortex and the inferior parietal lobe were activated during task duration performance and that both areas are also involved in the coding of the spatial properties of a task. Another example is the neurophysiological study performed with monkeys by Leon and Shadlen (2003). In this study, the authors showed that neurons in the posterior parietal cortex, an area involved in spatial processing, modulated their activity with the monkeys' perception of elapsed time (Gottlieb et al., 1998; Colby and Goldberg, 1999; Bisley and Goldberg, 2003). Moreover, deficits in both space and time are found after parietal damage (Critchley, 1953).

**Figure 1:**
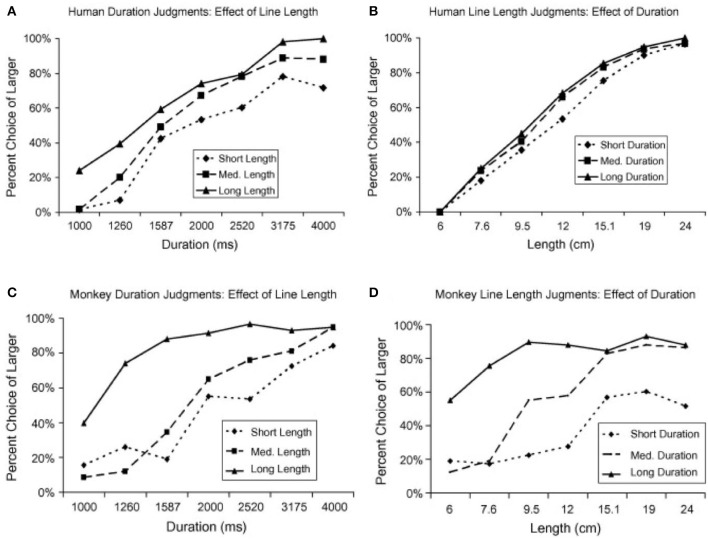
Humans and monkeys judgements of duration and line length (adapted from Merritt et al., 2010). **(A)** Humans estimations of stimulus duration (relevant variable) when crossed with three different values of line length (irrelevant variable): short (6 cm), medium (12 cm), and long (24 cm). **(B)** Humans estimations of line length (relevant variable) when crossed with three different values of duration (irrelevant variable): short (1,000 ms), medium (2,000 ms), and long (4,000 ms). **(C)** Same as in **(A)** but for monkeys estimations of duration. **(D)** Same as in **(B)** but for monkeys estimations of line length.

Additionally, Merritt et al. (2010) investigated whether the relationship between time and space was similar in humans and in non-human primates. Two rhesus monkeys and 16 adult humans had to classify the space or duration of a line displayed on a screen as long or short. Both magnitudes were presented but humans and monkeys had to attend to only one of them while the other was irrelevant. Humans showed an asymmetrical dependency between time and space, exhibiting a higher bias in their responses when they classified line durations as short or long (Figure 1A) than when they classified line lengths (Figure 1B). On the contrary, monkeys exhibited a large effect in their behavior of both, space on time (Figure 1C) and time on space (Figure 1D). One possible explanation for the differences in behavior between humans and monkeys might be due to the language capability, which is absent in monkeys. During linguistic communication, spatial terms are often used to refer to time and that might create an asymmetrical dependency between the two magnitudes even during non-linguistic scenarios. Another possibility is that humans, but not monkeys, are exposed to spatial representations of time through different technologies, such as, the horizontal line that indicates the elapsed and remaining time of a movie displayed on a computer.

## Space and time in the brain

Prefrontal and posterior parietal cortices play an important role during magnitude processing (Nieder et al., 2002, 2006; Dehaene et al., 2003b, 2004; Walsh, 2003; Nieder and Miller, 2004). Both areas have been extensively studied in a variety of spatial processing tasks in monkeys (Wilson et al., 1993; Rainer et al., 1998; Colby and Goldberg, 1999; Bisley and Goldberg, 2003) and humans (Astafiev et al., 2003; Merriam et al., 2003; Orban et al., 2004), as well as in time-interval judgements experiments (Onoe et al., 2001; Leon and Shadlen, 2003; Genovesio et al., 2006). Thus, a frontoparietal network of neurons seem to be involved in the representation of temporal and spatial information (Walsh, 2003; Buhusi and Meck, 2005; Merchant et al., 2013) and, interestingly, the same magnitude across different modalities, such as, the number of visual or auditory events, seems to be represented by the same neurons in the frontal lobe (Nieder, 2017). Moreover, modulation of the neural activity associated with timing has been found in the cerebellum (Perrett, 1998), basal ganglia (Jin et al., 2009), thalamus (Tanaka, 2007), posterior parietal cortex (Leon and Shadlen, 2003), prefrontal cortex (Brody et al., 2003; Genovesio et al., 2006, 2009, 2015; Oshio et al., 2008; Marcos et al., 2017), and premotor cortex (Lucchetti and Bon, 2001; Lebedev et al., 2008; Mita et al., 2009; Merchant et al., 2011; Confais et al., 2012).

**Figure 2:**
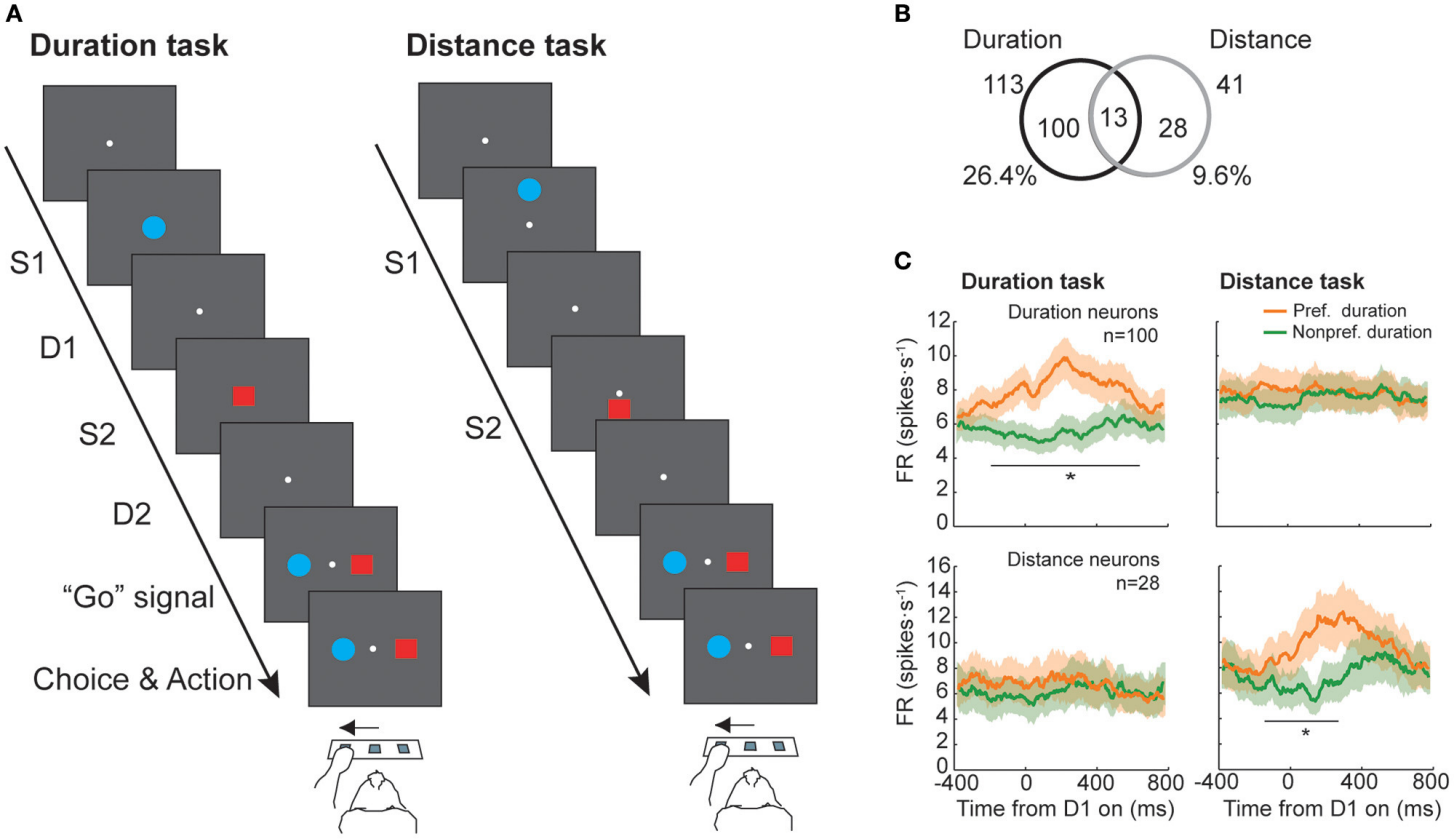
Duration and distance discrimination tasks (adapted from Marcos et al., 2017). **(A)** Temporal sequence of event during a trial. Each trial started with a pre-stimulus period in which monkeys were required to press and hold the central switch of the infrared array and was followed by the presentation of the first stimulus (S1). A delay period (D1) separated S1 offset from the presentation of the second stimulus (S2). After S1 offset, a second delay period (D2) preceded the reappearance of the two stimuli (targets), acting as “Go” signal, instructing the monkeys to choose the target that had either lasted longer, in the duration task, or had appeared farther from the central reference point, in the distance task. **(B)** Number of neurons with duration-related activity (*n* = 100), distance-related activity (*n* = 28) or duration- and distance-related activity (*n* = 13). **(C)** Mean firing rate for preferred and non-preferred conditions of the population of neurons previously classified as having duration- (Top panels) or distance-related (Bottom panels) modulation during the duration (Left panel) and distance (Right panel) tasks (^*^p < 0.05/24, paired-sample *t*-test with Bonferroni correction).

A neurophysiological study in macaques with recordings from neurons in the prefrontal cortex tested whether absolute duration and distance was coded by the same neural population (Marcos et al., 2017), testing whether a common magnitude's representation system exists in this area. Two monkeys were trained to perform two tasks: a duration and a distance discrimination task (Figure 2A). They had to select which of two stimuli, sequentially presented on a screen, had lasted longer or was presented farther from a reference point. The durations varied within the 200–1,200 ms range, in steps of 200 ms, while the range of distances changed from 8 to 48 mm, in steps of 8 mm, and were never the same for both stimuli. The performance of both monkeys was high, with a mean of 79 and 81% of correctly classified trials in the duration and the distance tasks, respectively (see also Genovesio et al., 2009, 2011 for further details). Neurons were classified based on their response during the delay period that followed S1 presentation (D1), because it is in this period when S1 duration could be determined. From the initial group of neurons recorded in the prefrontal cortex (*n* = 428), a total of 113 significantly represented long (1,000–1,200 ms) or short (200–400 ms) durations of S1 while 41 neurons coded long (40–48 mm) or short (8–16 mm) distances of S1. From the two groups only 13 neurons, 11.5 and 31.7% of the duration- and distance-related neurons, exhibited a significant modulation of their response for both magnitudes (Figure 2B). From this subgroup, 9 neurons showed a congruent preference across tasks, but the total number of overlapping neurons (13) was, however, not significant, indicating that the coding of duration and distance is performed independently in the prefrontal cortex. Consistent with this result, the mean firing rate of the two groups of neurons (113 and 41 neurons) showed a difference in response between preferred and non-preferred conditions for the magnitude that they significantly coded but not for the other (Figure 2C).

Similar to Marcos et al. (2017) and using the same experimental tasks, Genovesio et al. (2012) reported that neurons in prefrontal cortex also coded the relative duration and distance of the two stimuli in a domain-specific way. The relative magnitude was coded in the early decision period and was dissipated later. Interestingly, contrary to the high independency showed by the neurons representing the relative value of a specific magnitude, the great majority of neurons coded the non-spatial prospective goal (Rainer et al., 1999; Saito et al., 2005; Kusunoki et al., 2009)—the choice that will be subsequently selected (red or blue stimulus)—in a congruent way in both tasks. In particular, 92% of goal selective cells shared not only the same goal preference in the two tasks but also the same preference in a control matching to sample task that did not require any magnitude discrimination and that, therefore, allowed to unmistakably classify the neurons as goal selective. Consistent with the idea of goal coding, their goal selectivity persisted until the response could be provided. This result suggests that an interplay between magnitudes might occur in the prefrontal cortex at the level of goal choices rather than at the level of perceptual decisions.

Additionally, with the same dataset, Genovesio et al. (2016) found that neurons in the prefrontal cortex distinguished whether a delay interval (D1 and D2 in Figure 2A) was short or long in a context-dependent way. Therefore only a minority of the neurons coded the delays' durations in both tasks. Moreover, the authors also observed an independency in the neurons coding the first and second delay within a task showing that neurons distinguished between short and long durations not only across but also within tasks. Interestingly, when a neuron did code the elapsed duration of the two delays it did so in an uncorrelated or weakly correlated way. Such a context dependent timing representation suggest that prefrontal cortex keeps track of specific intervals. This specificity could help making foraging choices between patches of resources that take into account their relative locations, the effort to reach each patch and the relative amount of the resources (Genovesio et al., 2014). Similar high independency in the neural groups coding delays' durations was also observed for the representation of the relative duration of two stimuli (Genovesio et al., 2015). These results show a high context dependency in the representation of time in the prefrontal cortex. Rather than coding the time in an abstract manner, as a common timing mechanism, the neural representation of time in prefrontal cortex seems to be highly context dependent at the single cell level. Thus, neurons in prefrontal cortex do not only code magnitudes in an independent way but also the same magnitude in a context-specific way, supporting the hypothesis that in this area the interference between magnitudes probably occurs at the level of goal choices rather than at the level of magnitudes coding.

## Conclusions

Several studies have shown a clear interference between estimations of space and time. Spatial metaphors about time are often encountered in our daily communication and thoughts and are present also in the technological tools that we daily use (Lakoff and Johnson, 1980; Lakoff and Kovecses, 1987; McNeill, 1992). Interestingly, such interference between the two magnitudes emerges since our childhood (Casasanto et al., 2010). The neural mechanisms of that interference are, however, not fully understood. To shed light on this issue, several studies have focused on the neural overlap between brain areas during the perception or estimation of space and time, using functional neuroimaging techniques or through lesion studies. In these cases, a high overlap in the activation of specific brain areas during the estimation of both magnitudes have been observed suggesting a fronto-parietal network of neurons processing magnitudes in a general domain (Walsh, 2003; Buhusi and Meck, 2005; Merchant et al., 2013). Moreover, lesions of some brain areas influence the estimation of both space and time leading also to the idea of a general mechanism for magnitude processing (Critchley, 1953; Basso et al., 1996). However, these approaches allow only for a low spatial and temporal resolutions. A better resolution power can be obtained using neurophysiological techniques. Indeed, recent neurophysiological studies have investigated the coding of space and time at the single-cell level (Genovesio et al., 2012; Marcos et al., 2017). Neurons in the prefrontal cortex represent the absolute and relative values of distance and duration in a domain-specific way whereas, interestingly, they code the goal choices in a general manner. This opens the question of whether, at least in prefrontal cortex, in tasks with multiple domains the interference between magnitudes occurs during their perception and/or estimation or whether it occurs only in a subsequent step when the goal has to be chosen, as suggested by Genovesio et al. (2012). We cannot rule out the possibility that other parts of the brain, such as, the parietal cortex, can have domain-general representations considering that some parietal cortex neurons represent the same rule in both spatial and numerical domains (Eiselt and Nieder, 2013).

## Author contributions

All authors listed wrote the paper and have made a substantial, direct and intellectual contribution to the work, and approved it for publication.

### Conflict of interest statement

The authors declare that the research was conducted in the absence of any commercial or financial relationships that could be construed as a potential conflict of interest.
